# A Long-Term Disease-Free Case of Biphenotypic Sinonasal Sarcoma with Intracranial and Intraorbital Extension Initially Misdiagnosed as Synovial Sarcoma

**DOI:** 10.3390/diagnostics16020266

**Published:** 2026-01-14

**Authors:** Hiroyuki Morishita, Masayoshi Kobayashi, Masako Kitano, Kazuki Kanayama, Hiroshi Imai

**Affiliations:** 1 Department of Otorhinolaryngology-Head and Neck Surgery, Mie University Graduate School of Medicine, Tsu 514-8507, Mie, Japan; hiro-0707@med.mie-u.ac.jp (H.M.); machako@med.mie-u.ac.jp (M.K.); 2 Department of Oncologic Pathology, Mie University Graduate School of Medicine, Tsu 514-8507, Mie, Japan; stussy@suzuka-u.ac.jp; 3 Pathology Division, Mie University Graduate School of Medicine, Tsu 514-8507, Mie, Japan; qchan@med.mie-u.ac.jp

**Keywords:** biphenotypic sinonasal sarcoma, endoscopic endonasal approach, intracranial extension, low-grade sinonasal sarcoma with neural and myogenic differentiation, PAX3 rearrangement

## Abstract

Biphenotypic sinonasal sarcoma (BSNS) is a very rare, locally aggressive sarcoma arising in the sinonasal region, initially recognized as low-grade sinonasal sarcoma with neural and myogenic differentiation. Here, we report a case of BSNS extending into the intracranial and intraorbital regions, finally diagnosed by a break-apart fluorescence in situ hybridization (FISH) assay for rearrangements of *PAX3*. A 50-year-old woman presented with left diplopia and exophthalmos. CT and MRI revealed a large ethmoidal mass with intracranial and intraorbital extension. Since preoperative biopsy suggested a benign tumor, endoscopic endonasal resection was performed while preserving the anterior skull base and intraorbital structures. Postoperative histopathological diagnosis indicated synovial sarcoma, and proton beam therapy with adjuvant chemotherapy was subsequently administered. After treatment, FISH demonstrated rearrangements of *PAX3* and *MAML3* genes, leading to a revised diagnosis of BSNS, which typically does not require chemotherapy due to its non-metastatic behavior. Eleven years after treatment, the patient remains free of recurrence. Understanding BSNS is essential to avoid excessive intervention, and confirmation of *PAX3* rearrangement by FISH or equivalent molecular testing is crucial for accurate diagnosis.

**Figure 1:**
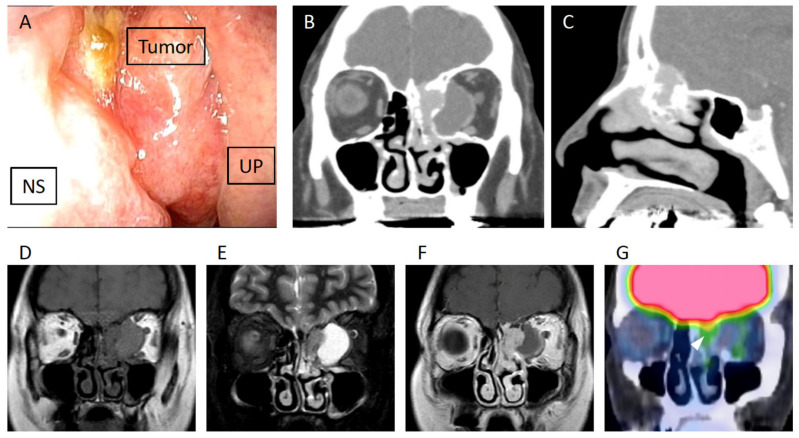
Preoperative endoscopic findings and imaging studies. A 50-year-old woman with a history of smoking one pack of cigarettes per day, a non-drinker, and with no prior medical illness, presented with a six-month history of left diplopia and exophthalmos. She was initially examined at an ophthalmology clinic and was subsequently referred to an otorhinolaryngologist in a general hospital, where CT revealed a sinonasal mass. A biopsy suggested a schwannoma and resulted in approximately 150 mL of hemorrhage at the outpatient clinic. The patient was then referred to our hospital for further evaluation and treatment. Nasal endoscopy revealed a smooth-surfaced tumor located in the middle meatus (**A**). NS: nasal septum; UP: uncinate process. Contrast-enhanced CT demonstrated a 30-mm mass consisting of a solid component within the ethmoid sinus and a cystic lesion extending into the orbit (**B**,**C**). The lesion involved the skull base and showed hyperostotic bone formation. MRI revealed that the solid portion of the mass had an isointense signal on T1- and T2-weighted images (**D**,**E**). On gadolinium-enhanced MRI, the lesion exhibited heterogeneous enhancement with thickening of the adjacent dura mater (**F**). These CT and MRI findings were not specific at the time of diagnosis; however, similar imaging features have been reported in biphenotypic sinonasal sarcoma (BSNS) in the literature. Positron emission tomography-computed tomography (PET-CT) showed a low fluorodeoxyglucose (FDG) uptake [the maximum standardized uptake value (SUVmax) = 2.2] ((**G**), white arrowhead). Review of the biopsy specimen by our pathologists revealed a spindle cell proliferation, suspicious for a spindle cell tumor, but it was difficult to determine whether it was benign or malignant.

**Figure 2:**
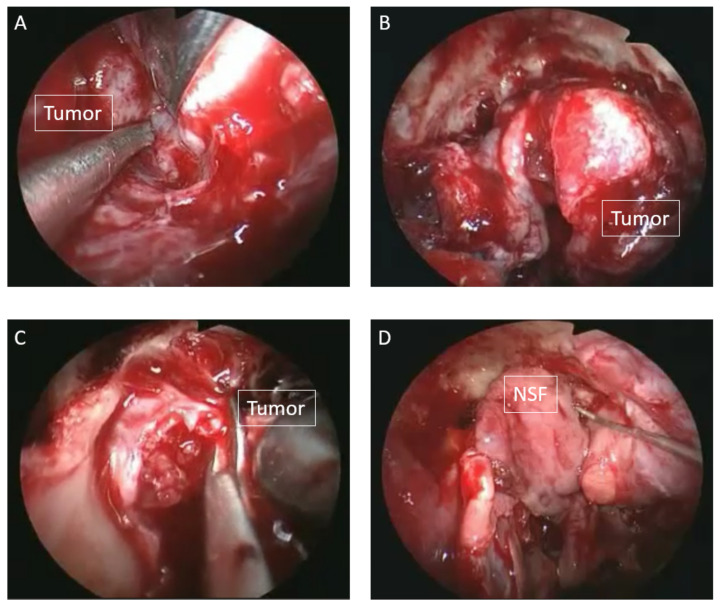
Intraoperative endoscopic findings. Since the preoperative diagnosis did not indicate malignancy, endoscopic endonasal resection was planned. First, an endoscopic modified Lothrop procedure (EMLP) was performed to secure an adequate surgical field, and the lateral portion of the tumor was detached from the orbital periosteum (**A**). As the tumor invaded the cribriform plate, it was circumferentially mobilized except for the superior aspect (**B**), and the involved cribriform plate and adherent dura mater were subsequently resected en bloc (**C**). No gross residual tumor was observed on the dura mater or arachnoid membrane surrounding the resection site. A small cerebrospinal fluid leak from an arachnoid fistula was repaired using a pedicled nasal septal mucosal flap (NSF) (**D**).

**Figure 3:**
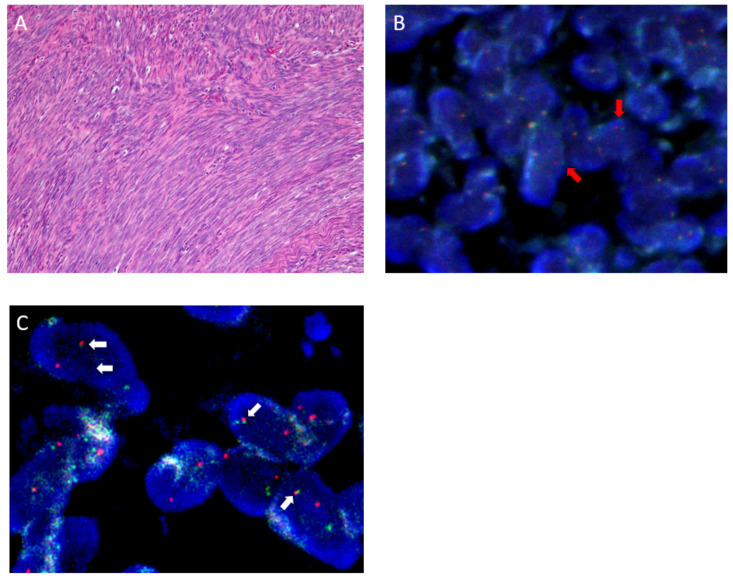
Pathological findings of the resected tumor. Postoperative histopathological examination revealed a monomorphic spindle cell proliferation without epithelial components (**A**). (**A**) shows a representative low-power histological section demonstrating uniform spindle cell morphology. Immunohistochemical staining was positive for BCL2, CD99/MIC2, S-100, cytokeratin AE1/AE3, and EMA, but negative for actin, CD56, and CD34. FISH using an SS18 break-apart probe demonstrated split red and green signals, indicating an SS18 rearrangement ((**B**), red arrows). (**B**) represents a representative fluorescence in situ hybridization (FISH) image. Accordingly, the tumor was diagnosed as monophasic synovial sarcoma. Given its malignant potential, adjuvant proton beam therapy (total 70.2 Gy) and three courses of doxorubicin plus ifosfamide chemotherapy were administered. One year later, in light of newly published reports on BSNS diagnosis [1], the pathological findings were re-evaluated. FISH using PAX3 and MAML3 fusion probes demonstrated overlapping red and green fusion signals confirming the PAX3-MAML3 fusion ((**C**), white arrows). (**C**) shows a representative FISH image demonstrating fusion signals, leading to a revised diagnosis of BSNS.

**Figure 4:**
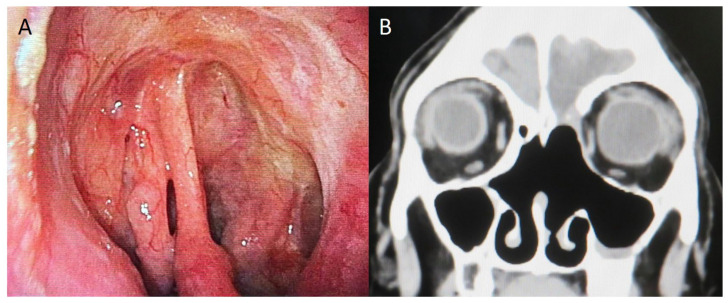
Endoscopic and imaging findings 11 years postoperatively. (**A**): Endoscopic view showing no evidence of recurrence in the nasal cavity. (**B**): Postoperative CT showing no findings suggestive of recurrence. Postoperative follow-up consisted of monthly outpatient visits during the first year, visits every 3 months from years 1 to 3, and visits every 6 months thereafter. Surveillance imaging with CT and MRI was performed every 4 months during the first postoperative year, every 6 months between years 2 and 5, and annually after 5 years. Since no suspicious findings were observed on endoscopic examination or imaging studies throughout the follow-up period, no biopsy or histological examination was performed. The patient has remained free of recurrence or metastasis for 11 years after surgery. BSNS is a very rare locally aggressive sarcoma arising in the sinonasal region and was initially recognized as low-grade sinonasal sarcoma with neural and myogenic differentiation [2]. Only a small number of clinical reports have been published since its first description in 2012 [1,3,4,5]. Because of its morphologic and immunophenotypic diversity, BSNS has often been misdiagnosed as various benign or malignant mesenchymal neoplasms [2,6]. The diagnosis has become more accurate since the discovery of the *PAX3* locus rearrangement, which is involved in the tumor’s pathogenesis [1]. Then, BSNS was newly included in the 2017 fourth edition of the World Health Organization Classification of Head and Neck Tumours [7]. There is a distinct female predominance (female-to-male ratio 2–3:1), and most affected individuals are in the fifties (age range: 24–87 years; mean 47 years) [6,8]. The nasal cavity (54%) and ethmoid sinus (57%) are the most commonly involved sites, either singly or in combination, and 25% of cases show orbital extension, while 11% involve the cribriform plate [2]. Intracranial extension, in particular, is uncommon, with only six cases reported to date [2,9,10,11]. Regarding prognosis, although almost half of the reported patients experienced local recurrence, none developed distant metastasis [1,2,9,11]. Only two tumor-related deaths have been documented, both of which occurred in cases with intracranial extension [9,10]. The present case was typical in terms of patient demographics and tumor localization, except for its invasion into the ethmoidal plate, dura mater, and orbit. The fact that this tumor exhibited intracranial extension but no recurrence represents a favorable long-term outcome. Preoperative diagnosis of BSNS is often challenging due to its rarity and histologic overlap with other spindle cell tumors. However, several characteristic imaging findings have been reported. A CT review of three cases demonstrated hyperostotic bone formation in all patients [11]. Another study reported that mixed sclerotic and lytic bone changes, with definitive hyperostosis identified in 80% (4 of 5 cases) [12]. On MRI, BSNS typically exhibits T2 signal intensity similar to cerebral gray matter and lower than that of most other sinonasal neoplasms; gadolinium enhancement is seen in all cases and is heterogeneous in 75% [12]. On PET-CT, the SUVmax of fluorodeoxyglucose is usually low, around 2.9, indicating borderline metabolic activity for sarcoma; therefore, PET findings are generally not helpful for diagnosis [13]. In the present case, CT demonstrated hyperostotic bone formation consistent with previous reports. MRI showed heterogeneous gadolinium enhancement, and PET-CT revealed a low FDG uptake (SUVmax 2.2), consistent with previously described BSNS characteristics. Recognition of these radiologic features might have allowed earlier consideration of BSNS in the differential diagnosis. Histologically, BSNS is characterized by a cellular spindle cell proliferation with uniform, elongated nuclei and an infiltrative growth pattern. While it may resemble adult fibrosarcoma or monophasic synovial sarcoma, BSNS shows a distinctive biphenotypic immunoprofile demonstrating both myogenic and neural differentiation [2]. However, available immunohistochemical markers are relatively nonspecific [14], which makes diagnosis difficult. A recurrent chromosomal translocation t(2;4)(q35;q31.1) has been reported to result in a *PAX3-MAML3* fusion protein, a potent transcriptional activator of the *PAX3* response element [1]. A previous study demonstrated that *PAX3* immunohistochemistry showed high sensitivity (100%) and specificity (98%) for BSNS in distinguishing it from histologic mimics such as malignant peripheral nerve sheath tumor, monophasic synovial sarcoma, spindle cell rhabdomyosarcoma, solitary fibrous tumor, sinonasal hemangiopericytoma, and cellular schwannoma [14]. Consequently, confirmation of *PAX3* locus rearrangement is considered essential for accurate diagnosis of BSNS. The present case was initially diagnosed as synovial sarcoma, but subsequent FISH revealed *PAX3* rearrangement, leading to the correct diagnosis of BSNS. From a clinical perspective, accurate initial diagnosis was crucial. If BSNS had been correctly diagnosed preoperatively, adjuvant chemotherapy could have been avoided, as no distant metastasis has ever been reported in BSNS, unlike synovial sarcoma, which has a postoperative distant recurrence rate of 39% [15]. To prevent unnecessary treatment, mesenchymal neoplasms of the sinonasal region should be carefully evaluated for *PAX3* rearrangement by molecular testing such as FISH. Compared with previously reported cases of biphenotypic sinonasal sarcoma with intracranial extension, which often showed local recurrence and occasionally tumor-related death [2,9,10,11], the present case demonstrated an exceptionally favorable long-term outcome with no recurrence or metastasis during an 11-year follow-up period. This case highlights the importance of accurate molecular diagnosis, as misclassification may lead to overtreatment [1].

## Data Availability

The original contributions presented in this study are included in the article. Further inquiries can be directed to the corresponding author.

## Abbreviations

The following abbreviations are used in this manuscript:

BSNS	biphenotypic sinonasal sarcoma
EMLP	endoscopic modified Lothrop procedure
FDG	fluorodeoxyglucose
FISH	fluorescence in situ hybridization
NS	nasal septum
NSF	nasal septal mucosal flap
PET-CT	positron emission tomography-computed tomography
SUVmax	maximum standardized uptake value
UP	uncinate process

## References

[1] Wang X., Bledsoe K.L., Graham R.P., Asmann Y.W., Viswanatha D.S., Lewis J.E., Lewis J.T., Chou M.M., Yaszemski M.J., Jen J. (2014). Recurrent PAX_3_-MAML_3_ fusion in biphenotypic sinonasal sarcoma. Nat. Genet..

[2] Lewis J.T., Oliveira A.M., Nascimento A.G., Schembri-Wismayer D., Moore E.A., Olsen K.D., Garcia J.G., Lonzo M.L., Lewis J.E. (2012). Low-grade sinonasal sarcoma with neural and myogenic features: A clinicopathologic analysis of 28 cases. Am. J. Surg. Pathol..

[3] Fritchie K.J., Jin L., Wang X., Graham R.P., Torbenson M.S., Lewis J.E., Rivera M., Garcia J.J., Schembri-Wismayer D.J., Westendorf J.J. (2016). Fusion gene profile of biphenotypic sinonasal sarcoma: An analysis of 44 cases. Histopathology.

[4] Kakkar A., Rajeshwari M., Sakthivel P., Sharma M.C., Sharma S.C. (2018). Biphenotypic sinonasal sarcoma: A series of six cases with evaluation of role of β-catenin immunohistochemistry in differential diagnosis. Ann. Diagn. Pathol..

[5] Le Loarer F., Laffont S., Lesluyes T., Tirode F., Antonescu C., Baglin A.C., Delespaul L., Soubeyran I., Hostein I., Pérot G. (2019). Clinicopathologic and molecular features of a series of 41 biphenotypic sinonasal sarcomas expanding their molecular spectrum. Am. J. Surg. Pathol..

[6] Gross J., Fritchie K. (2020). Soft tissue special issue: Biphenotypic sinonasal sarcoma: A review with emphasis on differential diagnosis. Head Neck Pathol..

[7] Stelow E.B., Bishop J.A. (2017). Update from the 4th Edition of the World Health Organization Classification of Head and Neck Tumours: Tumors of the nasal cavity, paranasal sinuses and skull base. Head Neck Pathol..

[8] Sethi S., Cody B., Farhat N.A., Pool M.D., Katabi N. (2021). Biphenotypic sinonasal sarcoma: Report of 3 cases with a review of the literature. Hum. Pathol..

[9] Rooper L.M., Huang S.C., Antonescu C.R., Westra W.H., Bishop J.A. (2016). Biphenotypic sinonasal sarcoma: An expanded immunoprofile including consistent nuclear β-catenin positivity and absence of SOX10 expression. Hum. Pathol..

[10] Lin Y., Liao B., Han A. (2017). Biphenotypic sinonasal sarcoma with diffuse infiltration and intracranial extension: A case report. Int. J. Clin. Exp. Pathol..

[11] Cannon R.B., Wiggins R.H., Witt B.L., Dundar Y., Johnston T.M., Hunt J.P. (2017). Imaging and outcomes for a new entity: Low-grade sinonasal sarcoma with neural and myogenic features. J. Neurol. Surg. Rep..

[12] Miglani A., Lal D., Weindling S.M., Wood C.P., Hoxworth J.M. (2019). Imaging characteristics and clinical outcomes of biphenotypic sinonasal sarcoma. Laryngoscope Investig. Otolaryngol..

[13] Powers K.A., Han L.M., Chiu A.G., Aly F.Z. (2015). Low-grade sinonasal sarcoma with neural and myogenic features-diagnostic challenge and pathogenic insight. Oral Surg. Oral Med. Oral Pathol. Oral Radiol..

[14] Jo V.Y., Mariño-Enríquez A., Fletcher C.D.M., Hornick J.L. (2018). Expression of PAX_3_ distinguishes biphenotypic sinonasal sarcoma from histologic mimics. Am. J. Surg. Pathol..

[15] Lewis J.J., Antonescu C.R., Leung D.H., Blumberg D., Healey J.H., Woodruff J.M., Brennan M.F. (2000). Synovial sarcoma: A multivariate analysis of prognostic factors in 112 patients with primary localized tumors of the extremity. J. Clin. Oncol..

